# A cross-sectional investigation of the ophthalmological impact of loiasis in Cameroon, Central Africa

**DOI:** 10.1371/journal.pntd.0013216

**Published:** 2025-06-26

**Authors:** Joseph Kamgno, Joseph Kamtchum-Tatuene, Linda Esso, Serge Bertrand Eyebe-Eyebe, Giles Kagmeni, Jean Bopda, Jean Tenaguem, Amy Klion, Hugues Nana-Djeunga, Lucienne Bella-Assumpta, Adrian Hopkins

**Affiliations:** 1 Higher Institute for Scientific and Medical Research (ISM), Yaounde, Cameroun; 2 Department of Public Health, Faculty of Medicine and Biomedical Sciences, University of Yaounde I, Yaounde, Cameroon; 3 Direction of Diseases, Epidemics and Pandemics Control, Ministry of Public Health, Yaounde, Cameroon; 4 Ophthalmology Unit, Yaounde University Teaching Hospital, Yaounde, Cameroon; 5 Department of Surgery and Surgical Specialties, Faculty of Medicine and Biomedical Sciences, University of Yaounde I, Yaounde, Cameroon; 6 Laboratory of Parasitic Diseases, National Institute of Allergy and Infectious Diseases, Bethesda, Maryland, United States of America; 7 Consultant, Blinding and Neglected Diseases of Poverty, Gravesend, United Kingdom; Zhejiang Wanli University, CHINA

## Abstract

**Background:**

Current knowledge of ocular manifestations of loiasis is limited to the transient subconjunctival passage of the adult filaria and anecdotal reports of posterior segment lesions. Therefore, the ocular burden of loiasis is likely underestimated since it has never been systematically assessed at the population level. We aimed to evaluate the relationship of *Loa loa* microfilaremia and recent eye worm passage with chronic ocular lesions identified through comprehensive ophthalmological assessment in an endemic area.

**Methodology/principal findings:**

Subjects aged ≥ 15 years, residing in Akonolinga for ≥ 5 years, without filaricidal treatment for ≥3 years, were screened for filariases. After excluding participants with onchocerciasis lesions, a subset of randomly selected participants was assessed by ophthalmologists blinded to blood test results then allocated to four groups defined by microfilarial load (MFL) on calibrated thick blood film: G1 (*Loa* MFL = 0), G2 (MFL < 8000/mL), G3 (MFL ≥ 8000/mL), G4 (co-infestation with *Mansonella* MFL > 100/mL). The ophthalmological assessment comprised distance visual acuity, examination of the anterior segment with a slit lamp, and fundoscopy. The primary analysis consisted of univariable comparisons of the frequency of abnormal findings across four groups (G1 – 4) or two groups defined by history of eye worm passage. The secondary analysis consisted of a multivariable logistic regression analysis of the relationship of high *Loa* MFL (≥8000/mL) with chorioretinitis and eye worm passage with unilateral ametropia, adjusting for confounders*.* Of 1511 subjects screened, 200 underwent ophthalmological assessment, including 65, 69, 35, and 16 in G1 to 4. History of eye worm passage in the previous year was reported by 121 participants (65.4%). Unilateral ametropia was more prevalent in people with history of eye worm passage in the previous year (26.5% versus 10.9%, p = 0.014). Chorioretinitis was the most frequent posterior segment lesion (n = 11, 6.1%) and was most prevalent in G3 (14.3%). The frequency of chorioretinitis was higher in participants with moderate-to-severe visual impairment (27.3% versus 4.4%, p = 0.002). High *Loa* MFL was an independent predictor of chorioretinitis (adjusted OR=5.28; p = 0.01). History of eye worm passage in the previous year was independently associated with unilateral ametropia (adjusted OR=3.27, p = 0.0088).

**Conclusions/significance:**

This study has, for the first time, provided evidence of independent association between history of eye worm passage and unilateral ametropia, and between high *Loa* MFL and severe chorioretinal lesions. This suggests that loiasis should be classified as a neglected tropical disease.

## Author summary

Loiasis is endemic in Western and Central Africa and affects more than 10 million individuals. Currents knowledge of its ocular manifestations is limited to the transient subconjunctival passage of the adult worm and anecdotal case reports of severe lesions such as optic neuritis and retinal hemorrhage. Consequently, loiasis has repeatedly failed to meet WHO’s morbidity and mortality criteria for inclusion in the strategic plan for neglected tropical diseases. We aimed to provide additional evidence to support this inclusion by studying the relationship of *Loa loa* microfilaremia and recent eye worm passage with chronic ocular lesions by performing a systematic ophthalmological assessment of community-dwelling adults in an endemic area. For the first time, we provide evidence that, contrary to prior knowledge, severe ocular lesions such as chorioretinitis are highly prevalent (14.3%) in people with high *Loa loa* microfilarial loads (MFL) and significantly associated with moderate-to-severe visual impairment. Specifically, people with a high MFL (>8000 mf/mL) are 5 times more likely to have chorioretinitis than those with low MFL or without infestation, independent of age, sex, co-infestation with other filaria*,* and cardiovascular risk factors. We also report that people with recent eye worm migration were 3 times more likely to have unilateral ametropia. Our findings contradict perceptions that loiasis is a benign disease and suggest that it should be classified as a neglected tropical disease. Such reclassification will boost the design and funding of cost-effective public health strategies to eliminate loiasis.

## Introduction

Loiasis is an African parasitic infection caused by the filarial nematode *Loa loa* and transmitted to humans by an insect vector called Chrysops [1,2]*.* It affects more than 10 million individuals and is associated with significant morbidity and mortality [3,4]. It is responsible for various systemic manifestations resulting from dermatologic [5–7], cardiac [8], renal [9], neurologic [10], gynecologic [11,12], and ophthalmological lesions [4,13]. Ocular manifestations described to date include orbital lesions caused by macrofilariae and posterior segment lesions caused by microfilariae. Anterior lesions refer to either the subconjunctival passage of the adult worm or orbital oedema, which both irritate but are not especially painful, the asymptomatic presence of an adult worm in the anterior chamber, or filarial uveitis [14]. Posterior segment lesions comprise retinal blood vessel embolism by microfilariae in people with high microfilarial loads [15], neuro-chorio-retinal lesions [16], retinal detachment [17], and retinal exudates or hemorrhages that occur spontaneously or as a consequence of antifilarial treatment [18].

Despite the manifestations described above, loiasis is generally considered a benign disease and is, therefore, neglected by health policy makers in endemic countries [7]. Loiasis is not even mentioned among neglected tropical diseases [19], which is likely a consequence of an underestimation of the true burden of its complications. Indeed, current knowledge of ocular manifestations of loiasis is mostly derived from case series or anecdotal case reports and no prior study has performed a systematic comprehensive ophthalmological assessment of community-dwelling individuals to detect chronic eye lesions potentially attributable to loiasis.

To bridge the knowledge gap, we aimed to determine the relationship between clinical and biological features of loiasis (eye worm passage, microfilarial load) and chronic ocular lesions in adults residing in a loiasis-endemic area. We hypothesized that eye lesions would be more prevalent in people with loiasis irrespective of their microfilarial load, that there would be a dose-response effect as a higher infestation burden could cause higher levels of systemic inflammation or higher risk of microvascular damage, and that co-infestation with *Mansonella perstans* could aggravate the deleterious effects of loiasis.

## Patients and methods

### Ethics statement

The study was approved by the Cameroon National Ethics Committee for Human Research (authorization number 044/CNE/MP/08), and participation was subject to signature of a written informed consent. For participants aged <18 years old, the written informed consent was obtained from the parent or legal guardian.

### Study site, design, and approval

This study was conducted in the Akonolinga Health District, which is located in the Centre Cameroon Forest approximately 100 km from the capital city Yaounde. The study site was chosen because it was predicted to be endemic for loiasis [20] and known to be hypo-endemic for onchocerciasis based on the rapid epidemiological mapping of onchocerciasis performed in 1997 [21]. These criteria were used to minimize onchocercal eye disease as a confounding factor when studying the relationship between ocular lesions and *Loa loa* microfilarial load.

The study comprised a screening phase, during which participants were assessed and selected based on their *Loa loa* microfilarial load, and a clinical phase focusing on comprehensive ophthalmological examination.

#### Phase 1: Screening for filariases.

All subjects aged 15 years and above, residing in the study area for at least five years, with no history of filaricidal treatment over the preceding three years, were invited to participate in the study. Communication about the study was performed several weeks prior to enrolment with the help of local authorities and community leaders. People willing to take part in the study were instructed to gather at one of five enrolment sites prepared by our research team in five out of 11 villages of the Akonolinga Health District, namely Abem, Edjom, Endom, Ekoudou, and Yeme Yeme. These villages were selected by random number generation. Enrolment sites were prepared either at the local health facility or at the village chief’s residence.

For each participant, we performed an interview, a physical examination, and a calibrated thick blood film. The interview aimed to collect demographic data (age, sex, place of birth, village of residence); successive places of residence since birth, and a history of prior symptoms consistent with loiasis (pruritus, Calabar swelling, subconjunctival *L. loa*, headache, arthralgia). The physical examination was performed by two experienced clinicians to identify lesions of onchocerciasis or lymphatic filariasis such as onchocercal nodules, hydrocele, and lymphoedema of the limbs, also called elephantiasis. Participants were also screened for hypertension and diabetes mellitus. Hypertension was defined as blood pressure > 140/90 mmHg on either or both arms [22]. Diabetes mellitus was defined as fasting blood glucose > 1.26 g/L.

The calibrated thick blood film (CTBF) was performed between 11 a.m. and 3 p.m. using whole blood freshly drawn using 70-µL non-heparinized microhematocrit capillary tubes. Thick blood smears were prepared by spraying 50 µL of whole blood on a glass slide to form a rectangle of 3 by 2 centimeters. The smears were dried, dehemoglobinized for 10–15 minutes using buffered water, fixated with methanol, then stained for 30 minutes with Giemsa solution diluted 1/10. After further rinsing and drying, slides where read under a microscope (10X objective) to identify and count microfilariae of *Loa* loa (270–300 µm by 6–8 μm, sheathed, tapered tail with nuclei extending to the tip) and *Mansonella perstans* (smaller, 180–240 µm by 3–5 μm, and unsheathed). All slides were read by two experienced parasitologists at the laboratory of the Higher Institute for Scientific and Medical Research, Yaounde, Cameroon. In case of discrepancy, a third independent reader was involved.

#### Phase 2: Ophthalmological assessment.

After excluding participants with onchocercal nodules or lesions of lymphatic filariasis, and those who had resided in an area endemic for onchocerciasis for at least two years, 200 subjects were randomly selected to undergo an ophthalmological examination by consultant ophthalmologists blinded to CTBF results. The random sampling without replacement from the screening dataset was performed with SPSS 12.0 (International Business Machines Corporation, New York, USA).

The ophthalmological examination comprised (i) assessment of distance visual acuity using an E illiterate chart and a pin hole for gross correction in patients with visual acuity less than 1, (ii) examination of the anterior segment of the eye using a portable slit lamp (Kowa Company Ltd, Nagoya, Japan), and (iii) examination of the posterior segment of the eye using either a slit lamp with an indirect 78-diopter lens (Volk optical inc., Ohio, USA) or an ophthalmoscope (HEINE Optotechnik GmbH, Gilching, Germany). Ophthalmological examinations were conducted in health centers or in a dedicated room provided by a community leader. In this field epidemiology study, refractive error was defined as a visual acuity less than 10/10 in at least one eye that improves by at least 1 point after using the pinhole. Moderate-to-severe visual impairment was defined as best corrected visual acuity less than 3/10 for both eyes, as per the World Health Organization criteria [23].

All participants diagnosed with retinal lesions underwent retinal angiography and a skin snip test to rule out onchocerciasis. All angiographies were performed at the Ophthalmology Unit of the Yaounde Central Hospital, while the skin snip tests were performed by experienced biologists at the Higher Institute for Scientific and Medical Research.

### Data analysis

Categorical and continuous data were summarized as count (proportion) or median (range) unless otherwise stated.

**Fig 1:**
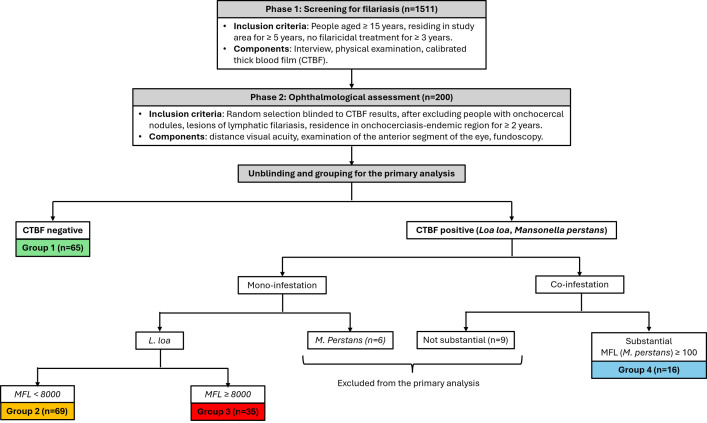
Study design. CTBF: Calibrated thick blood film, MFL: microfilarial load (mf/mL).

In the primary analysis, we performed various comparisons between four groups defined by CTBF results to investigate the ophthalmological impact of loiasis and the potential modulating effect of the co-infestation with *Mansonella perstans* (Fig 1). Group 1 comprised participants with negative CTBF. Participants with mono-infestation by *Loa loa* were assigned to Group 2 (low microfilarial load, i.e., < 8000 microfilariae of *Loa loa* per milliliter of blood, mf/mL) or Group 3 (high microfilarial load, ≥ 8000 mf/mL). The threshold of 8000 mf/ml was chosen because patients harboring more than 8000 *L. loa* mf/ml are at higher risk of severe adverse reactions after treatment by ivermectin [24]. Group 4 comprised participants co-infested with *L. loa* and *M. perstans* with at least 100 mf/ml of *M. perstans* (substantial co-infestation). Participants with mono-infestation by *Mansonella perstans* and those with non-substantial co-infestation (less than 100 mf/ml of *M. perstans* irrespective of their *Loa loa* microfilarial load) were excluded from the primary analysis (n = 15). Participants were considered positive for a given anterior or posterior segment condition if characteristic lesions were observed in either or both eyes. We also performed additional analysis to identify factors associated with eye worm passage and explore its potential consequences on eye health. Fisher’s exact test was used to compare proportions of participants with a specific clinical, biological or ophthalmological condition between groups defined by microfilarial load of *Loa loa* and *Mansonella perstans* or by history of eye worm passage. Kruskal-Wallis test was used to compare medians of continuous parameters between groups.

In the secondary analysis, that included all participants in Phase 2, we performed a multivariable binary logistic regression analysis of the association between chorioretinitis and high microfilarial loads of *Loa loa* (≥8000 mf/mL irrespective of the co-infestation with *Mansonella perstans*), while adjusting for age (continuous), sex, hypertension, diabetes mellitus, tobacco consumption, history of eye worm passage during the previous year, and presence of infestation by *Mansonella perstans*. We also performed a multivariable binary logistic regression analysis of the association between history of eye worm passage during the previous year and unilateral ametropia, while adjusting for high microfilarial loads of *Loa loa,* age (continuous), sex, hypertension, diabetes mellitus, tobacco consumption, and presence of infestation by *Mansonella perstans*. In the multivariable regression analyses, all variables listed above were forced into the model and no a priori selection was applied because our aim was not to build a perfect prediction model for chorioretinitis or unilateral ametropia, but rather to test if their respective association with high *Loa loa* microfilarial load or history of eye worm passage is robust to the adjustment for all potential confounders. Variables were only excluded in case of collinearity, that is if they were perfect combinations of one another. The significance of individual regression coefficients was determined by a Wald test. The goodness of fit of the multivariable regression model was assessed with the Hosmer-Lemeshow chi-squared test. The model performance was assessed by the percent explained variance (R^2^); the model calibration by calculating the proportion of observations correctly classified, and the model discrimination by computing the area under the receiver operating characteristic curve (AUC).

Sensitivity analyses were performed to verify the specificity of the association between high *Loa loa* microfilarial load and chorioretinitis. First, we refitted the multivariable logistic regression with *Loa loa microfilaremia* as the primary independent variable while adjusting for presence of infestation with *Mansonella perstans*, age (continuous), sex, hypertension, diabetes mellitus, tobacco consumption, and history of eye worm passage during the previous year. Second, we refitted the multivariable logistic regression with any microfilaremia as the primary independent variable while adjusting for *Loa-Mansonella* coinfestation, age (continuous), sex, hypertension, diabetes mellitus, tobacco consumption, and history of eye worm passage during the previous year.

All statistical tests were two-sided, and the significance threshold was defined as p ≤ 0.05. Data were analyzed with SPSS 12.0 (International Business Machines Corporation, New York, USA).

## Results

We screened 1511 subjects (median age: 50 years, range: 15.0 – 91.0), and performed ophthalmological assessment in a subset of 200 randomly selected participants including 65 in Group 1, 69 in Group 2, 35 in Group 3 and 16 in Group 4 (Fig 1 and Table 1). Fundoscopy was not possible in nine subjects due to bilateral lens opacity (three in Group 1, one in Group 2, one in Group 4, two with *M. perstans* mono-infestation, and two with non-substantial co-infestation). Among participants undergoing ophthalmological assessment, 44 patients had a high microfilarial load of *Loa loa* including 35 with *Loa loa* mono-infestation (Group 3), six with substantial co-infestation (Group 4), and three with non-substantial co-infestation (excluded from the primary analysis). Fundoscopy was not possible in 2 subjects with high microfilarial load due to bilateral lens opacity (one in Group 4 and one with non-substantial co-infestation).

**Table 1:** Clinical characteristics of participants.

Characteristic^a^	Group 1 (n = 65)	Group 2 (n = 69)	Group 3 (n = 35)	Group 4 (n = 16)	p
	Negative calibrated thick blood film	*Loa loa microfilarial load <8000 mf/mL*	*Loa loa microfilarial load* ≥*8000 mf/mL*	Substantial co-infestation with *Loa loa* and *Mansonella perstans*	
Age (years), median (range)	56 (21-84)	57 (15-88)	59 (15-80)	64 (33-74)	0.28
Female	47 (72.3)	34 (49.3)	15 (42.9)	5 (31.3)	0.0021
Hypertension	34 (52.3)	29 (42.0)	16 (45.7)	7 (43.8)	0.69
Diabetes mellitus ^b^	10 (15.4)	9 (13.4)	1 (2.9)	1 (6.3)	0.24
Tobacco consumption	24 (36.9)	24 (34.7)	16 (45.7)	11 (68.8)	0.076
Eye worm passage in previous year	41 (63.1)	47 (68.1)	26 (74.3)	7 (43.8)	0.19
Episodes of eye worm passage in previous year, median (range)	3 (1-12)	3 (1-12)	3 (2-15)	3 (1-12)	0.77
More than 10 episodes of eye worm passage in previous year	4 (6.2)	7 (10.1)	4 (11.4)	2 (12.5)	0.68
Moderate-to-severe visual impairment ^c^	6 (9.2)	3 (4.3)	2 (5.7)	3 (18.8)	0.23
Microfilarial load (mf/mL), median (range)					
*Loa Loa*	–	1380 (20-7640)	18480 (8300-98380)	2820 (20-88180)	–
*Mansonella perstans*	–	–	–	300 (120-10300)	–

^a^Categorical variables are presented as count (percent).

^b^Missing value for diabetes mellitus in 2 participants from Group 2.

^c^Defined as best corrected visual acuity ≤ 3/10 for both eyes as per the World Health Organization criteria.

In the primary analysis, 121 participants (65.4%) had had at least one episode of eye worm passage during the year preceding the study and 18 participants (9.7%) reported having 10 episodes or more, with no difference between the four groups defined by presence and density of microfilaremia (Table 1). Unilateral ametropia was more prevalent in people with a history of eye worm passage in the previous year (26.5% versus 10.9%, p = 0.014, Table 2). There was a trend towards a higher number of participants with more than 10 episodes of eye worm passage in the previous year in people with unilateral ametropia (41.2% versus 19.1%, p = 0.055). None of the patients with ametropia (unilateral or bilateral) had visual impairment.

**Table 2:** Univariable analysis of factors associated with eye worm passage in the previous year for participants included in the primary analysis (n = 185).

	Eye worm passage in previous year		p
	Yes (n = 121)	No (n = 64)	
**Clinical characteristics** ^a^			
Age (years), median (range)	55 (15-88)	60 (15-84)	0.28
Female	67 (55.4)	34 (53.1)	0.88
Hypertension	55 (45.5)	31 (48.4)	0.76
Diabetes mellitus^b^	16 (13.5)	5 (7.8)	0.33
Tobacco consumption	48 (39.7)	27 (42.2)	0.76
Moderate-to-severe visual impairment	8 (6.6)	6 (9.4)	0.56
**Biological parameters**			
Positive calibrated thick blood film			
*Loa Loa*	80 (66.1)	40 (62.5)	0.63
*Mansonella perstans*	7 (5.8)	9 (14.1)	0.096
Microfilarial load (mf/mL), median (range)			
*Loa Loa*	860 (0-98380)	190 (0-67040)	0.35
*Mansonella perstans*	0 (0-840)	0 (0-10300)	0.35
High *Loa loa* microfilarial load	28 (23.1)	13 (20.31)	0.71
**Findings on ophthalmological exam**			
Refractive errors			
*Unilateral*	32 (26.5)	7 (10.9)	0.014
*Bilateral*	7 (5.8)	8 (12.5)	0.16
*Any*	39 (32.3)	15 (23.4)	0.23
Anterior segment lesions			
*Cataract*	31 (25.6)	13 (20.3)	0.47
*Sub-conjunctival calcified worm*	2 (1.7)	0 (0.0)	0.55
*Pterygium*	13 (10.7)	6 (9.4)	0.99
Posterior segment lesions^c^			
*Optic atrophy*	2 (1.7)	2 (3.3)	0.61
*Chorioretinitis*	8 (6.7)	3 (4.9)	0.75
*Retinal hemorrhage*	1 (0.8)	0 (0.0)	0.99
*Vascular retinopathy*	3 (2.5)	2 (3.3)	0.99
*Age-related macular degeneration*	1 (0.8)	0 (0.0)	0.99

^a^Categorical variables are presented as count (percent).

^b^Missing value for two participants with positive history of eye worm passage.

^c^Data are from 180 out of 185 participants included in the primary analysis. Fundoscopy was not possible in 2 participants with history of eye worm passage and 3 without.

There was no difference in the frequency of refractive errors, moderate-to-severe visual impairment, and anterior segment lesions between the four groups defined by presence and density of microfilaremia (Table 3). Chorioretinitis was the most frequent lesion on posterior segment examination (11 cases, 6.1%), and it was more prevalent in group 3 versus groups 1, 2, and 4 (14.3% versus 8.1%, 0%, and 6.7%; p = 0.01) (Table 3). There was only a 7% power to detect the nearly two-fold difference in frequency of chorioretinitis between group 3 and group 1. Chorioretinitis was bilateral in 4 cases, including 3 in group 3 and 1 in group 4. Bilateral chorioretinitis was more frequent in group 3 versus group 1 (8.6% versus 0.0%, p = 0.044), and significantly more prevalent in people with high versus low *Loa loa* microfilarial load (9.5% versus 0.0%, p = 0.0021). None of the retinal lesions found had the characteristics of toxoplasmic chorioretinitis. There were no cases of moderate-to-severe visual impairment or chorioretinitis in participants excluded from the primary analysis.

**Table 3:** Distribution of abnormal findings on ophthalmological assessment in groups defined by *Loa loa* microfilarial load.

Findings	Group 1 (n = 65)	Group 2 (n = 69)	Group 3 (n = 35)	Group 4 (n = 16)	Total	p
	Negative calibrated thick blood film	*Loa loa microfilarial load <8000 mf/mL*	*Loa loa microfilarial load* ≥*8000 mf/mL*	Substantial co-infestation with *Loa loa* and *Mansonella perstans*		
**Refractive errors**						
*Unilateral*	12 (18.5)	16 (23.2)	8 (22.9)	3 (18.8)	39 (21.1)	0.92
*Bilateral*	5 (7.7)	7 (10.1)	1 (2.9)	2 (12.5)	15 (8.1)	0.502
*Any*	17 (26.2)	23 (33.3)	9 (25.7)	5 (31.3)	54 (29.2)	0.77
**Anterior segment lesions**						
*Cataract*	14 (21.5)	15 (21.7)	10 (28.6)	5 (31.3)	44 (23.8)	0.71
*Sub-conjunctival calcified worm*	0 (0)	0 (0)	2 (5.7)	0 (0)	2 (1.1)	0.075
*Pterygium*	8 (12.3)	6 (8.7)	1 (2.9)	4 (25.0)	19 (10.3)	0.096
**Posterior segment lesions** ^a^						
*Optic atrophy*	2 (3.2)	1 (1.5)	1 (2.9)	0 (0)	4 (2.2)	0.88
*Chorioretinitis*	5 (8.1)	0 (0)	5 (14.3)	1 (6.7)	11 (6.1)	0.011
*Retinal hemorrhage*	0 (0)	0 (0)	1 (2.9)	0 (0)	1 (0.6)	0.28
*Vascular retinopathy*	1 (1.6)	2 (2.9)	0 (0)	2 (13.3)	5 (2.8)	0.093
*Age-related macular degeneration*	0 (0)	0 (0)	0 (0)	1 (6.7)	1 (0.6)	0.083

^a^Fundoscopy was not possible for three participants in Group 1, one participant in Group 2 and one participant in Group 4.

**Fig 2:**
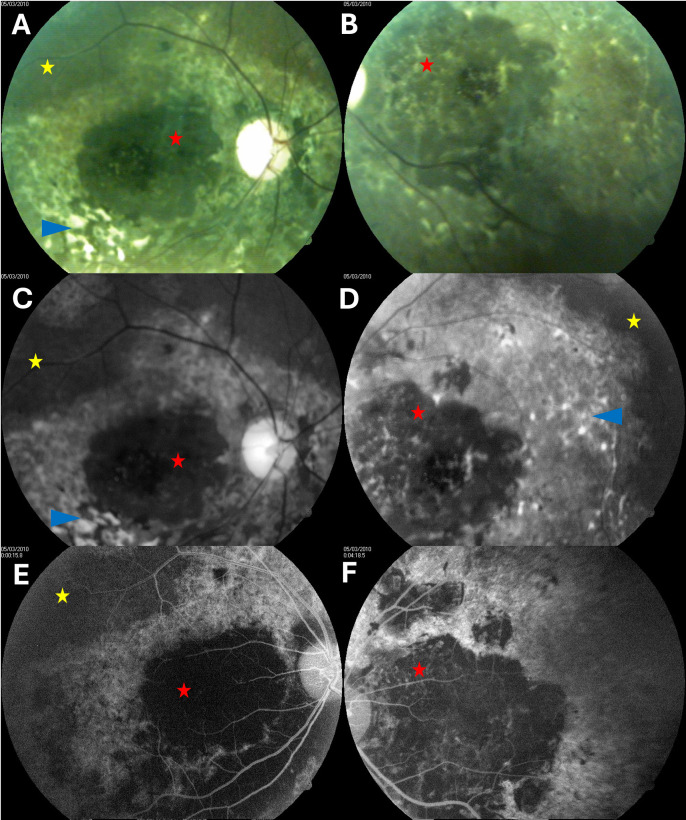
Fundus findings in a 47-year-old woman with a *Loa loa* microfilarial load of 88180 mf/mL, a negative skin snip test, and no vascular risk factors. Right eye is shown in the left column and left eye in the right column. Color fundus photographs (A, B), green light photographs (C, D), and late-phase fluorescein angiogram (E, F) show large macular chorioretinal scar with central presumed subfoveolar fibrosis (red stars), concentric areas of abnormal retinal pigment epithelium in the posterior pole (blue arrowheads), peripheral retinal pigment epithelium atrophy (yellow stars), and diffuse vascular thinning.

The biological characteristics of participants with chorioretinitis are presented in Table 4. Two of them had possible concomitant onchocerciasis with 2 and 0.5 mf per skin biopsy, while only one had co-infestation with *Loa loa* and *Mansonella perstans*. Angiography results for the 47-year-old female participant with chorioretinitis who had the highest *Loa loa* microfilarial load are presented in Fig 2. The distribution of the other posterior segment lesions did not differ between the groups. Only one case of retinal hemorrhage was found in a 69-year-old nondiabetic male with a *Loa loa* microfilarial load of 11380 mf/ml. He was also found to have stage II high blood pressure (207/98 mmHg).

**Table 4:** Clinical and biological characteristics of participants with chorioretinitis.

#	Sex	Age	Study group	Hypertension	Diabetes	Tobacco consumption	Episodes of eye worm in previous year	Visual impairment	Retinal lesions	Microfilarial load		
										*L. loa* (mf/mL)	*M. perstans* (mf/mL)	*O. volvulus* (mf/2 biopsies)^a^
1	Male	44	3	No	No	Yes	2	No	Unilateral	67840	0	4
2	Male	60	3	Yes	No	Yes	0	Yes	Bilateral	10520	0	0
3	Male	61	3	No	No	Yes	2	No	Bilateral	44540	0	1
4	Male	64	1	No	No	Yes	12	No	Unilateral	0	0	0
5	Female	47	4	No	No	No	2	Yes	Bilateral	88180	120	0
6	Female	50	3	Yes	No	Yes	3	No	Unilateral	10660	0	0
7	Female	57	1	No	No	Yes	0	No	Unilateral	0	0	0
8	Female	67	1	Yes	No	No	8	No	Unilateral	0	0	0
9	Female	67	1	Yes	No	No	6	No	Unilateral	0	0	0
10	Female	69	1	No	No	No	8	No	Unilateral	0	0	0
11	Female	73	3	No	No	No	0	No	Bilateral	0	0	0

^a^A molecular test was not performed on the microfilariae found on skin snip tests to formally establish whether they were *Onchocerca* species.

In the secondary analysis, the frequency of chorioretinitis was higher in participants with moderate-to-severe visual impairment (27.3% versus 4.4%, p = 0.018), and in participants with a *Loa loa* microfilarial load >8000 mf/mL (14.3% versus 3.4%, p = 0.016). In the multivariable binary logistic regression, a high microfilarial load of *Loa loa* was the single independent predictor of chorioretinitis (adjusted OR=5.28; 95% CI: 1.42-19.57, p = 0.013, Table 5). In the sensitivity analyses, there was no association between chorioretinitis and *Loa loa* microfilaremia or any microfilaremia (S1 and S2 Tables). A history of eye worm passage in the previous year was independently associated with unilateral ametropia (adjusted OR=3.27, 95% CI: 1.35-7.93, p = 0.0088, Table 6).

**Table 5:** Univariable and multivariable analysis of factors associated with chorioretinitis.

Potential predictors ^a^	Univariable model		Multivariable model	
	OR (95% CI)	p	OR (95% CI)	p
*Loa loa* microfilarial load >8000 mf/mL	4.8 (1.39-16.62)	0.013	5.46 (1.44-20.59)	0.012
Age (years)	1.03 (0.98-1.07)	0.25	1.04 (0.99-1.09)	0.12
Female	1.53 (0.43-5.41)	0.51	2.42 (0.61-9.64)	0.21
Hypertension	0.97 (0.29-3.31)	0.97	0.60 (0.15-2.33)	0.46
Tobacco consumption	1.76 (0.52-5.98)	0.37	1.62 (0.44-5.92)	0.47
Infestation with *Mansonella perstans*	0.62 (0.08-5.06)	0.66	0.58 (0.06-5.27)	0.63
Eye worm passage in previous year	1.44 (0.37-5.60)	0.603	1.67 (0.38-7.23)	0.49

Hosmer-Lemeshow chi-squared test for goodness of fit of the multivariable model: p = 0.85. Area under the Receiver Operating Characteristic curve (AUC) = 0.80. Count R^2^ = 93.4%. Proportion of patients correctly classified = 93.4%.

^a^Diabetes mellitus was omitted because of collinearity. All participants with chorioretinitis were diabetes-free.

**Table 6:** Univariable and multivariable analysis of factors associated with unilateral ametropia.

Potential predictors	Univariable model		Multivariable model	
	OR (95% CI)	p	OR (95% CI)	p
Eye worm passage in previous year	2.89 (1.26-6.66)	0.012	3.27 (1.35-7.93)	0.0088
*Loa loa* microfilarial load >8000 mf/mL	0.80 (0.34-1.88)	0.604	0.91 (0.36-2.28)	0.84
Age (years)	1.04 (1.01-1.06)	0.002	1.04 (1.02-1.08)	0.0026
Female	1.47 (0.73-2.95)	0.28	1.38 (0.64-2.99)	0.41
Hypertension	1.44 (0.73-2.86)	0.29	0.81 (0.37-1.78)	0.61
Diabetes	1.94 (0.73-5.12)	0.18	1.62 (0.56-4.69)	0.38
Tobacco consumption	2.03 (1.02-4.04)	0.043	1.99 (0.94-4.2)	0.07
Infestation with *Mansonella perstans*	0.89 (0.34-2.33)	0.81	1.00 (0.34-2.92)	0.99

Hosmer-Lemeshow chi-squared test for goodness of fit of the multivariable model: p = 0.23. Area under the Receiver Operating Characteristic curve (AUC) = 0.73. Count R^2^ = 80.2%. Proportion of patients correctly classified = 80.2%.

## Discussion

This study provides evidence that high microfilarial loads of *Loa loa* are independently associated with chorioretinitis lesions, suggesting that onchocerciasis is not the only filariasis responsible for visual impairment. Indeed, we made reasonable efforts to exclude cases of onchocerciasis by (i) selecting a study site not endemic for onchocerciasis, (ii) excluding participants harboring onchocercal nodules, and (iii) excluding people who lived in an area endemic for onchocerciasis for two years or more. We did not find microfilariae of *O. volvulus* on slit lamp examination of the anterior segment in any of the subjects selected for the second phase of the study. Moreover, we did not find lesions suggestive of ocular onchocerciasis such as uveitis, sclerosing keratitis, peripapillary ring-shaped atrophy with hypo-autofluorescence, and macula-sparing pigment epithelial atrophy affecting the temporal and nasal retina [25–27]. Findings of other epidemiological studies in Central Africa also support the potential contribution of loiasis to posterior segment lesions. For instance, in Ogooué-Lolo province in Gabon, a region hypoendemic for onchocerciasis (7%), Fobi *et al*. reported a low prevalence of anterior segment lesions and a high prevalence of optic nerve (5.2%) and chorio-retinal lesions (2.7%) [28], which could be related to the high prevalence of loiasis (27.9%) [29].

Among participants with chorioretinal lesions, only two were found to have positive skin snip tests with very low onchocercal microfilarial loads. Both subjects also had very high loads of *Loa loa* microfilariae (44540 and 67840 mf/mL), which could have been detected on the skin snip tests leading to the positive results, as previously described [30]. The chorioretinal lesions observed on fundoscopy could be scars of repeated retinal hemorrhages or after-effects of microembolisation of retinal blood vessels by microfilariae in cases of massive infestation [15]. Indeed, the unique case of retinal hemorrhage was reported in a participant with a high microfilarial load (11380 mf/mL), albeit there was a concurrent diagnosis of stage II high blood pressure. Although the reported chorioretinal lesions have some overlapping features with geographical atrophy seen in age-related maculopathy, the lack of association with age and cardiovascular risk factors makes the diagnosis unlikely, especially in participants younger than 50 years [31,32].

A history of eye worm passage was highly frequent in our study population. In 2002, Takougang *et al.* carried out a survey simultaneously in Cameroon and Nigeria and demonstrated that a 40% prevalence of history of eye worm passage corresponds to a 20% prevalence of *Loa loa* microfilaremia [33]. The results obtained in the Akonolinga health district are, therefore, consistent with the conclusions of their study, since we found a 23% prevalence of *Loa loa* microfilaremia [34]. There was no difference between study groups with respect to the frequency of a history of eye worm passage, which confirms the low sensitivity of CTBF as a diagnostic tool for loiasis [35,36]. It also emphasizes the absence of a relationship between *Loa loa* microfilarial load and the frequency of eye worm passages. Indeed, we also found no difference between the groups with respect to the mean annual number of eye worm passages per individual. This is explained, at least in part, by the fact that the number of adult worms is independent of the *Loa loa* microfilarial load [6,37].

We report an independent association between eye worm passage in the previous year and unilateral ametropia. The underlying reason for this association is not straightforward and it may be a chance finding. However, one could hypothesize that the eye worm passage induces an edema of periorbital and ocular tissues, especially the cornea. This edema alters the refractive properties of the affected eye resulting in unilateral ametropia. In theory, the ametropia would be transient, resolving when the edema disappears, and leaving no permanent effect on visual acuity [4,38]. But it is possible that repeated passages induce more permanent or irreversible damage. This hypothesis would align with the reported trend towards a higher number of participants with more than 10 episodes of eye worm passage in the previous year among individuals with unilateral ametropia. Unfortunately, we could not explore this further in our data analysis because we did not record the side of the most recent eye worm passage to check if it matches the side of the ametropia and the time elapsed since the event to assess if a certain degree of edema was likely to be present. Moreover, we did not follow participants to ascertain if some cases of ametropia would be reversible. Further studies are, therefore, needed to confirm our findings.

Despite the large sample size and the robust methodology used to establish the ophthalmological impact of loiasis, our study has some limitations. First, most patients with loiasis are amicrofilaremic and Giemsa-stained thick blood smear has low diagnostic sensitivity in individuals with low microfilarial loads [39,40]. Therefore, it is possible that some individuals with very low microfilarial loads were misclassified in group 1, thus leading to attenuation of the association between loiasis and choriretinitis. Using polymerase chain reaction-based assays could have increased our statistical power by limiting the extent of such potential classification bias. Unfortunately, molecular tests were not affordable at the time of the study. Moreover, we did not perform a molecular test on the microfilariae identified on skin snip tests and could not test for IgG4 antibodies against *Onchocerca volvulus-*specific antigen Ov16 to formally rule out any history of onchocerciasis in people with chorioretinal lesions [30,41]. Therefore, although the probability of onchocerciasis is low, it remains possible that some cases of chorioretinitis identified were attributable to onchocerciasis. Second, we did not assess color vision during the ophthalmological examination and cannot comment on either the potential relationship of loiasis with impaired color vision or the consequences of the observed chorioretinal lesions on color vision in affected subjects. Third, we did not perform optical coherence tomography to further characterize the retinal lesions and better understand the potential underlying pathobiological mechanism. Fourth, we did not collect longitudinal data to assess the incidence of new chorioretinal lesions in people with high microfilarial loads versus controls as well as the evolution of refraction errors. Consequently, further studies are needed to confirm the link between loiasis, chorioretinal lesions and visual impairment.

In conclusion, this is the first study performing a systematic ophthalmological assessment of community-dwelling adults to provide evidence that, contrary to previous knowledge, loiasis is associated with chronic ophthalmological lesions that could lead to visual impairment. This supports the idea that loiasis should be classified as a neglected tropical disease. Novel efficient and cost-effective public health strategies to eliminate loiasis are, therefore, urgently needed.

## Supporting information

S1 Table Univariable and multivariable analysis of factors associated with chorioretinitis using any microfilaremia as the main independent variable. (PDF)

S2 Table Univariable and multivariable analysis of factors associated with chorioretinitis using *Loa loa* microfilaremia as the main independent variable. (PDF)

S1 Data Dictionary Explanatory table for the variables included in the database. (PDF)

S1 Database Study data. (XLSX)
