# Supplementary material for: A cross-sectional investigation of the ophthalmological impact of loiasis in Cameroon, Central Africa
Source: PLoS Negl Trop Dis. 2025 Jun 26;19(6):e0013216. doi: 10.1371/journal.pntd.0013216 (PMC12225979; doi:10.1371/journal.pntd.0013216)
Supplement: S1 Table — (PDF) [file pntd.0013216.s001.pdf]

S1 Table: Univariable and multivariable analysis of factors associated with chorioretinitis

Potential predictors [a]	Univariable model		Multivariable model	
	**OR (95% CI)**	**p**	**OR (95% CI)**	**p**
Presence of any microfilaremia *(Loa loa or Mansonella perstans*)	0.56 (0.17-1.90)	0.35	0.56 (0.14-2.17)	0.39
Age (years)	1.03 (0.98-1.07)	0.25	1.03 (0.98-1.08)	0.19
Female	1.53 (0.43-5.41)	0.51	1.56 (0.40-5.99)	0.52
Hypertension	0.97 (0.29-3.31)	0.97	0.63 (0.17-2.37)	0.49
Tobacco consumption	1.76 (0.52-5.98)	0.37	1.82 (0.51-6.50)	0.35
Loa-Mansonella coinfestation	0.76 (0.09-6.22)	0.79	0.88 (0.09-8.57)	0.92
Eye worm passage in previous 12 months	1.44 (0.37-5.60)	0.603	1.46 (0.36-5.98)	0.59

Hosmer-Lemeshow chi-squared test for goodness of fit of the multivariable model: p = 0.53. Area under the Receiver Operating Characteristic curve (AUC) = 0.70. Count R^2 = 93.4%. Proportion of patients correctly classified = 93.4%.

[a] Diabetes mellitus was omitted because of collinearity. All participants with chorioretinitis were diabetes-free.
