# Supplementary material for: A cross-sectional investigation of the ophthalmological impact of loiasis in Cameroon, Central Africa
Source: PLoS Negl Trop Dis. 2025 Jun 26;19(6):e0013216. doi: 10.1371/journal.pntd.0013216 (PMC12225979; doi:10.1371/journal.pntd.0013216)
Supplement: S2 Table — (PDF) [file pntd.0013216.s002.pdf]

S2 Table: Univariable and multivariable analysis of factors associated with chorioretinitis

Potential predictors [a]	Univariable model		Multivariable model	
	**OR (95% CI)**	**p**	**OR (95% CI)**	**p**
Presence of *Loa loa* microfilaremia	0.62 (0.18-2.10)	0.44	0.65 (0.17-2.44)	0.52
Age (years)	1.03 (0.98-1.07)	0.25	1.03 (0.98-1.08)	0.19
Female	1.53 (0.43-5.41)	0.51	1.57 (0.41-6.04)	0.51
Hypertension	0.97 (0.29-3.31)	0.96	0.64 (0.17-2.40)	0.51
Tobacco consumption	1.76 (0.52-5.98)	0.37	1.85 (0.52-6.59)	0.34
Infection with *Mansonella perstans*	0.62 (0.08-5.06)	0.66	0.61 (0.07-5.44)	0.66
Eye worm passage in previous 12 months	1.44 (0.37-5.60)	0.603	1.44 (0.35-5.82)	0.61

Hosmer-Lemeshow chi-squared test for goodness of fit of the multivariable model: $p = 0.50$. Area under the Receiver Operating Characteristic curve (AUC) = 0.70. Count R^2 = 93.4%. Proportion of patients correctly classified = 93.4%.

[a] Diabetes mellitus was omitted because of collinearity. All participants with chorioretinitis were diabetes-free.
