# Supplementary material for: A cross-sectional investigation of the ophthalmological impact of loiasis in Cameroon, Central Africa
Source: PLoS Negl Trop Dis. 2025 Jun 26;19(6):e0013216. doi: 10.1371/journal.pntd.0013216 (PMC12225979; doi:10.1371/journal.pntd.0013216)
Supplement: S1 Data Dictionary — (PDF) [file pntd.0013216.s003.pdf]

Data dictionary

Variable name	Variable label	Values	Value labels
Idn	Unique identification number	Integers	NA
Age	Age of the participant (years)	Integers	NA
Sex	Sex of the participant	1, 2	Male, Female
Eyewormhistory	History of eye worm passage in the previous 12 months	Integers	NA
Counteyeworm12	Number of episodes of eye worm passage in the past 12 months	Integers	NA
Tas_bg_mmhg	Systolic blood pressure, left arm (mmHg)	Integers	NA
Tad_bg_mmHg	Diastolic blood pressure, left arm (mmHg)	Integers	NA
Tas_bd_mmhg	Systolic blood pressure, right arm (mmHg)	Integers	NA
Tad_bd_mmHg	Diastolic blood pressure, right arm (mmHg)	Integers	NA
Fbs	Fasting blood sugar (mmol/L)	Decimals	NA
Tobacco	Smoking	0, 1	No, Yes
Lfod	Lesions of anterior segment on slit lamp examination, right eye	0 to 9	0= No lesion<br>1= Cataract<br>2= Calcified worm under the conjunctiva<br>3= Mobile adult worm<br>4= Corneal opacity<br>5= Corneal ulceration<br>6= Corneal dryness<br>7= Conjunctival hemorrhage<br>8= Pterygium<br>9= Other lesions
Lfog	Lesions of anterior segment on slit lamp examination, left eye		
Fod	Findings on fundoscopy, right eye	0 to 9	0= No lesion<br>1= Optic atrophy<br>2= Chorioretinitis<br>3= Age-related macular degeneration<br>4= Chorioretinal degeneration<br>5= Retinal hemorrhage<br>6= Vascular retinopathy<br>7= Fundoscopy not possible<br>8= Papillary edema<br>9= Other lesions
Fog	Findings on fundoscopy, left eye		
Chargloa	Microfilarial load, *Loa loa*, mf/mL	Integers	NA
Chargmp	Microfilarial load, *Mansonella perstans*, mf/mL	Integers	NA
avodsc	Distant visual acuity, right eye, no correction	Integers (over 10)	NA
Avodts	Distant visual acuity, right eye, pinhole correction	Integers (over 10)	NA
avogsc	Distant visual acuity, left eye, no correction	Integers (over 10)	NA
Avogts	Distant visual acuity, left eye, pinhole correction	Integers (over 10)	NA
Fopossible	Fundoscopy possible	0, 1	No, Yes
